# RT-SHIV subpopulation dynamics in infected macaques during anti-HIV therapy

**DOI:** 10.1186/1742-4690-6-101

**Published:** 2009-11-04

**Authors:** Wei Shao, Mary Kearney, Frank Maldarelli, John W Mellors, Robert M Stephens, Jeffrey D Lifson, Vineet N KewalRamani, Zandrea Ambrose, John M Coffin, Sarah E Palmer

**Affiliations:** 1 Advanced Biomedical Computing Center, SAIC Frederick, Inc, National Cancer Institute at Frederick, Frederick, MD, USA; 2 HIV Drug Resistance Program, NCI, Frederick, MD, USA; 3 Division of Infectious Diseases, University of Pittsburgh School of Medicine, Pittsburgh, PA, USA; 4 AIDS and Cancer Virus Program, SAIC Frederick, Inc, National Cancer Institute at Frederick, Frederick, MD, USA; 5 Tufts University, Boston, MA, USA; 6 Department of Virology, Swedish Institute for Infectious Disease Control and Karolinska Institutet, Stockholm, Sweden

## Abstract

**Background:**

To study the dynamics of wild-type and drug-resistant HIV-1 RT variants, we developed a methodology that follows the fates of individual genomes over time within the viral quasispecies. Single genome sequences were obtained from 3 pigtail macaques infected with a recombinant simian immunodeficiency virus containing the RT coding region from HIV-1 (RT-SHIV) and treated with short-course efavirenz monotherapy 13 weeks post-infection followed by daily combination antiretroviral therapy (ART) beginning at week 17. Bioinformatics tools were constructed to trace individual genomes from the beginning of infection to the end of the treatment.

**Results:**

A well characterized challenge RT-SHIV inoculum was used to infect three monkeys. The RT-SHIV inoculum had 9 variant subpopulations and the dominant subpopulation accounted for 80% of the total genomes. In two of the three monkeys, the inoculated wild-type virus was rapidly replaced by new wild type variants. By week 13, the original dominant subpopulation in the inoculum was replaced by new dominant subpopulations, followed by emergence of variants carrying known NNRTI resistance mutations. However, during ART, virus subpopulations containing resistance mutations did not outgrow the wide-type subpopulations until a minor subpopulation carrying linked drug resistance mutations (K103N/M184I) emerged. We observed that persistent viremia during ART is primarily made up of wild type subpopulations. We also found that subpopulations carrying the V75L mutation, not known to be associated with NNRTI resistance, emerged initially in week 13 in two macaques. Eventually, all subpopulations from these two macaques carried the V75L mutation.

**Conclusion:**

This study quantitatively describes virus evolution and population dynamics patterns in an animal model. The fact that wild type subpopulations remained as dominant subpopulations during ART treatment suggests that the presence or absence of at least some known drug resistant mutations may not greatly affect virus replication capacity *in vivo*. Additionally, the emergence and prevalence of V75L indicates that this mutation may provide the virus a selective advantage, perhaps escaping the host immure system surveillance. Our new method to quantitatively analyze viral population dynamics enabled us to observe the relative competitiveness and adaption of different viral variants and provided a valuable tool for studying HIV subpopulation emergence, persistence, and decline during ART.

## Background

Antiretroviral therapy (ART) suppresses HIV-1 replication *in vivo *but does not eradicate the virus. Consequentially, drug resistance remains a major obstacle to effective therapy [1]. Recent evidence indicates that mucosal transmission of HIV-1 infection usually involves the establishment of systemic infection by only a single viral variant [2-5]. After transmission, the virus is able to diversify into complex subpopulations due to its rapid replication cycle and high mutation rate. In a ten year period, HIV-1 genomes in an infected patient can be 3000 generations removed from the initial infecting virus [1]. Understanding HIV population dynamics and evolution is therefore important for understanding AIDS pathogenesis and the emergence of drug resistance mutations [6,7].

The intra-patient evolution of HIV-1 subpopulations can be shaped by several selective forces, including host immune surveillance, ART, and competition between different virus variants for host resources [8,9]. A major factor affecting HIV-1 evolution in treated patients is the emergence of drug resistant mutations, which have been reported for all effective antiviral drugs developed to date [10]. Mutations conferring escape from both humoral and cellular immune responses are also frequent [11,12]. To date, there have been few longitudinal studies on the dynamics of virus subpopulations within infected individuals, including their emergence, persistence, prevalence, and decline during infection and treatment. Charpentier *et al*. followed the emergence of drug resistance mutations in patients treated with protease inhibitors and described the dynamics of the major HIV-1 subpopulations [13]. Ball *et al *proposed a mathematical model to describe intra-host HIV evolution in terms of mutation, competition, and strain replacement [14,15]. However, quantitative documentation of virus population structure and dynamics during the course of infection is rare in the literature. One particular difficulty with HIV-1 in infected patients is that the virus population structure at the time of infection, and shortly thereafter, cannot be directly assessed. For this reason, we have analyzed plasma from macaques infected with a well-defined SIV chimeric virus containing the RT coding region of HIV-1 (RT-SHIV_mne_) [16]. In an earlier study, we reported the frequency of drug resistance mutations in virus isolated from longitudinal plasma samples after infection and during treatment [17]. We report here the analysis of multiple single genome sequences to quantify the number of subpopulations (populations consisting of identical virus variants) and to analyze the complex dynamics of these populations during the course of infection and treatment.

## Results

### Population structures in early stages of RT-SHIV infection and treatment of animal M03250

HIV-1 RT subpopulation dynamics were analyzed in the plasma of 3 pigtail macaques infected with RT-SHIV (Table 1). Samples were obtained from a previous study aimed at evaluating the effects of prior exposure to NNRTI monotherapy on subsequent combination ART [17], similar to the use of single-dose nevirapine to prevent mother to child transmission [18-21]. The animals were treated with a short course of efavirenz (EFV) at week 13, followed by daily combination therapy of tenofovir (TNF), emtracitabine (FTC), and EFV from weeks 17-37 post-inoculation. Frequent and convenient sampling, access to the virus inoculum, and lack of adherence issues make the RT-SHIV macaque model ideal for investigating viral population dynamics prior to initiating therapy, after initiating short-course monotherapy, and during ART.

**Table 1:** Treatment and sampling intervals for the 3 macaques.

Number of samples^a^				EFV	←	ART							→		
**Week of sampling**	**0**	**1**	**12**	**13^b^**	**17**	**17.5**	**19**	**22**	**23**	**24**	**25**	**26**	**37**	**39**	**40**

M03250	39	24		37	40	44	41	35	33	37	43	41			
M04007	39	12		20	38										32
M04008	39	33		23	41				19					31	

^a ^Number of sequences obtained at each time point.

^b ^Week 13 was sampled before EFV treatment.

**Figure 1:**
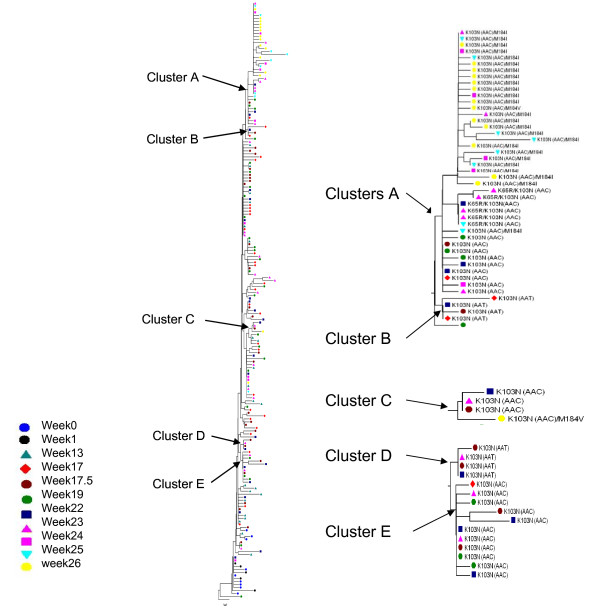
**Phylogenetic analysis of RT-SHIV subpopulations of macaque M03250**. The left panel is a neighbor-joining tree of all subpopulations of M03250. Each subpopulation is shown as a single sequence for this tree construction. Subpopulations from each week are represented by symbols coded with the same color and shape. The internal nodes from which subpopulation clusters containing drug resistant mutations appeared are marked as clusters A, B, C, D, and E, shown enlarged to the right.

Macaque M03250 failed the combination therapy with the appearance of multidrug resistant virus starting at week 22, 5 weeks after combination ART was initiated. Viremia in the other two macaques remained suppressed during the course of therapy. In each virus population, dominant and minor subpopulations were found among the sequences obtained by single-genome sequencing (SGS) at the time points shown in Table 1. The sequence of each subpopulation of M03250 was used to construct a neighbor-joining tree (Figure 1), with subpopulations from the same week labeled with a symbol of the same color and shape and each subpopulation represented by a leaf in the tree. In this animal, RT-SHIV evolved into a very complex population in which subpopulations from early time points persisted over the course of infection, while other subpopulations were lost. Subpopulations containing the drug resistance mutations K103N (AAC and AAT) formed 5 clusters in the phylogeny (Figure 1 Clusters A-E), indicating that they emerged independently. The earliest subpopulations containing the EFV resistance mutation K103N were observed at week 17 in both clusters A (AAA to AAC)) and B (AAA to AAT)) and at week 17.5 in clusters C, D, and E (Figure 1).

**Figure 2:**
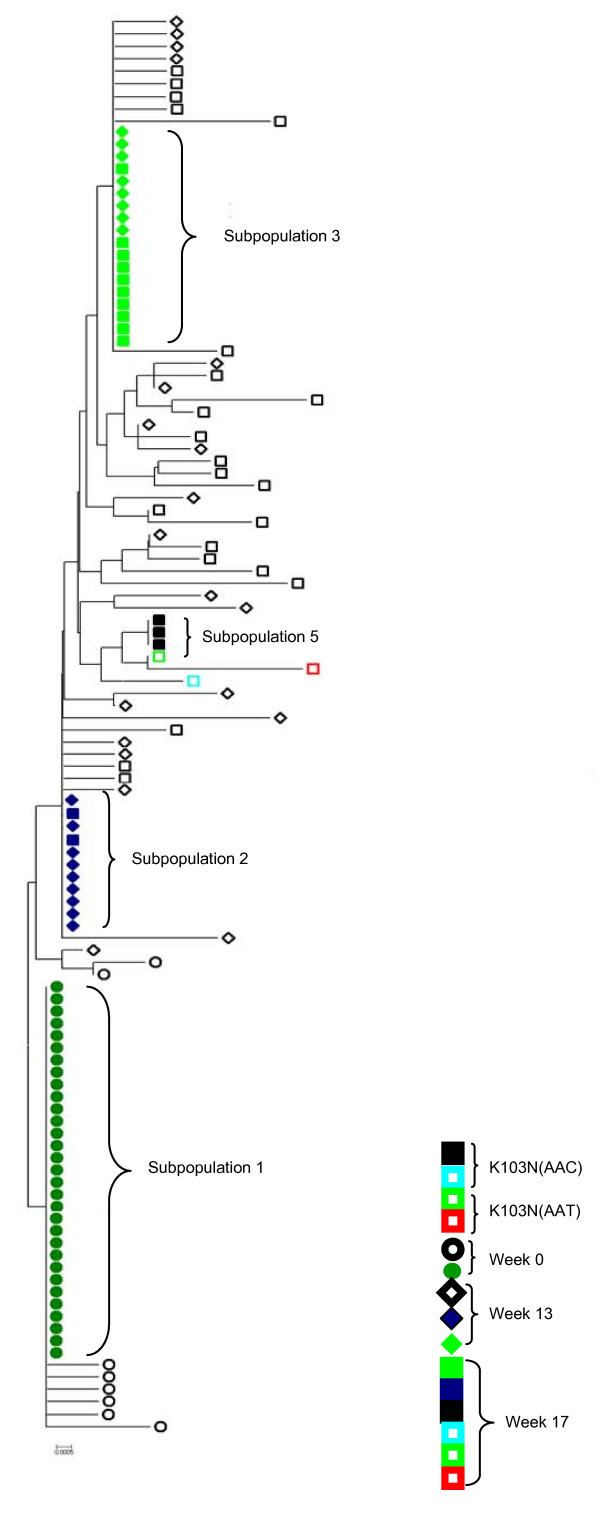
**Structure of RT-SHIV populations from macaque M03250**. The tree shows sequences from weeks 0 (circles), 13 (diamonds), and 17 (squares). Subpopulations consisting of multiple sequences are marked with solid shapes while subpopulations consisting of single sequence are marked with hollow shapes. Different subpopulations containing K103N are also labeled with solid shapes. Subpopulation designations (subpopulations 1, 2, 3, 5, and 6) correspond to those in Figures 3 and 4. Subpopulations not shown in Figures 3 and 4 were not given a subpopulation designation.

Neighbor joining trees were also constructed from all sequences obtained for each time point. Figure 2 shows the RT-SHIV population from week 0, week 13 (just prior to EFV monotherapy), and week 17 from monkey M03250. Several distinct subpopulations were evident, some consisting of only one sequence with others comprising multiple identical sequences (up to 10). At week 0, there was one dominant subpopulation (subpopulation 1, solid dark green circles). At week 13, the virus population was characterized by two dominant subpopulations, (subpopulations 2 and 3) each comprising 24% of the total population (solid green diamonds and solid blue diamonds) while the remaining 52% comprised minor subpopulations of unique sequences (Figure 2, hollow diamonds). However, at week 17 following EFV monotherapy, there was only one dominant subpopulation (subpopulation 3, solid light green squares), although two members of subpopulation 2 were still present. At the same time, 6 variants containing K103N (AAC) or K103N (AAT) were detected, which formed 4 subpopulations. Subpopulation 5 (solid black squares) comprised 3 virus sequences while the other 3 each had only one sequence (hollow colored squares, Figure 2). In all samples analyzed from each of the infected monkeys, we consistently found one or two dominant subpopulations, along with many minor subpopulations.

### Subpopulation dynamics in monkey M03250

**Figure 3:**
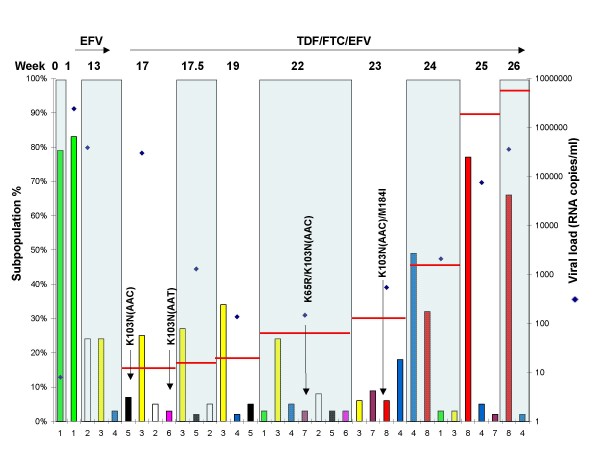
**Dynamics of major subpopulations in macaque M03250**. The major subpopulations present at the weeks shown on the x-axis are represented by colored bars. Subpopulations with identical sequences at different times have the same number and color and drug resistant populations are labeled. The left y-axis shows the percentage of a subpopulation in the total population. The right y-axis shows the viral load (diamond symbols) for the whole population at each week. Treatments are shown with arrows at the top of the figure along with the week post-infection (week 0 denotes the RT-SHIV challenge stock). Horizontal red bars show the percentage of all subpopulations with drug resistant mutations (K65R, K103N, M184I, and M184V) in the population of each week.

**Figure 4:**
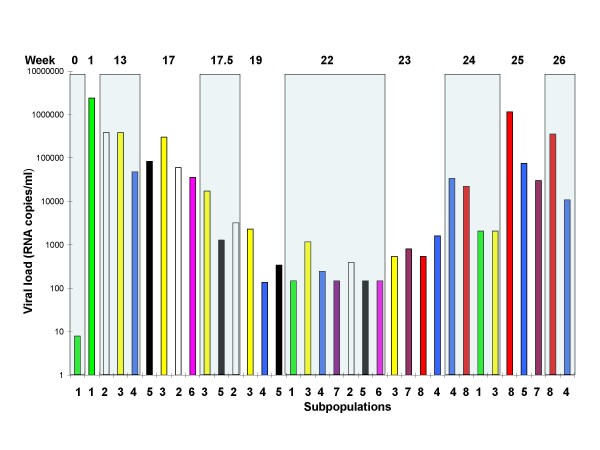
**Dynamics of major subpopulations expressed as normalized viral load from macaque M03250**. The y-axis shows the normalized viral load (frequency × viral RNA copies/ml plasma) of a subpopulation. The subpopulation designation and color code are the same as shown in Figure 3.

Figure 3 shows the fates of selected RT-SHIV subpopulations in M03250, expressed as percentages of the whole viral population at each sampling week and Figure 4 shows the same subpopulations as viral RNA copies/ml plasma. As shown in Figures 3 and 4, the dominant subpopulation found in the original virus challenge stock (sub1, week 0) was also the dominant subpopulation in the first plasma sample collected from M03250 (Figure 3, sub 1 at week 0 and week 1). This variant, however, was not found by week 13 as new subpopulations emerged. It was replaced by two new wild type dominant subpopulations emerging at week 13 prior to EFV treatment (sub2, 24% and sub3, 24%; Figure 3). The frequency of sub2 declined significantly between weeks 13 and 17, and the two remained relatively constant throughout a 5 weeks period on combination therapy and a 3-log decline in viremia, even though neither subpopulation carried any known drug resistant mutation. They subsequently became minor species at weeks 23 and 24 (Figure 3).

During the course of infection and treatment, many subpopulations carrying drug resistant mutations emerged. However, none became dominant before the emergence and expansion (to about 75% of the virus population) of the double mutant K103N/M184I (resistant to both EFV and FTC), beginning at week 23 and coincident with the onset of virologic failure. EFV resistance mutations (K103N) initially were observed at week 17, the first sample after EFV monotherapy: an AAC allele (sub5, Figures 3 and 4) and an AAT allele (sub6, Figures 3 and 4). The AAC subpopulation remained minor until week 22, after which time it became undetectable. The AAT subpopulation was detected at week 17 and never became dominant. The same was true at weeks 22 and 23 for a variant carrying two drug resistance mutations: K65R/K103N(AAC) (Figure 3, sub7), encoding resistance to TNF as well as EFV. Overall, at week 23, 6 weeks after the initiation of ART, 11 out of 23 subpopulations contained K103N and 4 out of 23 subpopulations contained K65R (3 as K65R/K103N). Subpopulations with a single K103N mutation (without linkage to another drug resistance mutation) were 30% of all viral populations of week 23 (Additional file 1) and none was the dominant subpopulation (Figure 3). In the neighbor joining tree, subpopulations containing K103N(AAC)/K65R or K103N(AAC)/M184I and several others containing K103N(AAC) formed a cluster. Several subpopulations containing K103N(AAT) and K103N(AAC) formed another cluster (Figure 1). In total, 5 of 9 subpopulations contained K103N at week 24, all existing as minor subpopulations. The subpopulations containing only K103N were 2.7% of the population at this week, declining from 30.1% at week 23 (Additional file 1), a result of the takeover by the doubly resistant subpopulation 8.

The subpopulation that led to virologic failure in this macaque carried the linked drug resistant mutations K103N(AAC)/M184I. This species was observed at week 23 as two subpopulations: week 23-26 (sub8) and week 23-32 (Additional file 1), one becoming undetectable the very next week (week 23-32 in Additional file 1), and one persisting and leading to virologic failure at weeks 25 and 26 (sub8 in Figures 3 and 4). Interestingly, a wild-type subpopulation first appeared as a minor species at week 13 (Figure 3, sub4) and became dominant (50%) after failure of combination therapy in week 24. This subpopulation did not carry any drug resistance mutations, yet it persisted and even increased about 9-fold in frequency and 300-fold in terms of its absolute amount over the course of infection and treatment through week 26 (Figures 3 and 4).

Another mutation not associated with drug resistance at position 75 in RT (V75L) was detected first in every subpopulation at week 13 in animal M03250 prior to EFV treatment. This mutation was also present in almost all subpopulations at later time points (Additional file 1). It was not detected by SGS in the challenge stock used to infect this macaque and was not seen at week 1. It was possible that this variant existed in the challenge stock at a frequency below our detection sensitivity by SGS. Therefore, we used 454 pyrosequencing to increase the sensitivity of detecting the V75L mutation in the viral inoculum. We obtained 10,836 *pol *sequences from the virus challenge stock by 454 pyrosequencing and none contained the V75L mutation (data not shown). This data show that V75L emerged after inoculation.

In addition, we observed changes in the frequency of a polymorphic allele (L/F) at position 214. The challenge stock and all viral variants at week 1 had 100% L at this position. At week 13, about 41% of variants had 214F and its frequency increased each week thereafter, reaching 100% at week 25 and 93% at week 26 (Additional file 1).

### Subpopulation dynamics in macaque M04008

**Figure 5:**
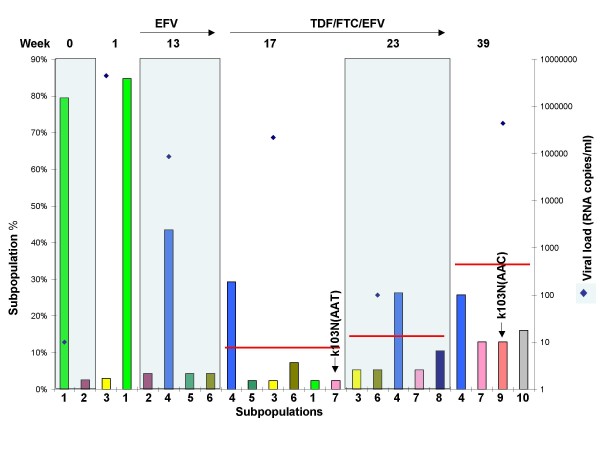
**Dynamics of major subpopulations in macaque M04008**. The major subpopulations present at the weeks shown on the x-axis are represented by colored bars. Subpopulations with identical sequences at different times have the same number and color and drug resistance mutations are labeled. The left y-axis shows the percentage of a subpopulation in the total population. The right y-axis shows the viral load (diamond symbols) for the whole population of each week. Treatments are shown with arrows at the top of the figure along with the week post-infection (week 0 denotes the RT-SHIV challenge stock). Horizontal red bars show the percentage of all subpopulations with drug resistant mutations (K65R, K103N, M184I, and M184V) in the population of each week.

Macaque M04008 received the same ART treatment regimen as M03250. Again, drug resistant mutations first appeared as minor subpopulations at week 17, following short course EFV monotherapy. In contrast to M03250, none led to virologic failure on combination therapy, although their overall frequency at week 17 was similar in the two macaques (Additional files 1 and 2). As in M03250, the original wild type subpopulation (Figure 5, sub1) was also dominant one week after infection. It declined to a minor subpopulation in subsequent samples. Another wild type subpopulation replaced it as the dominant subpopulation in week 13 (Figure 5, sub4). In this macaque, several subpopulations carrying drug resistant mutations emerged during treatment. For example, 5 of the 17 minor subpopulations carried K103N by week 17 (Additional file 2). At week 39, 5 of 14 subpopulations carried K103N (3 with AAT and 2 with AAC), together accounting for about 35% of the total sequences obtained at week 39 (Additional file 2). The initial K103N subpopulations in M04008 did not persist, but rather new variants containing K103N appeared and replaced previous subpopulations. For example, only one minor subpopulation with K103N at week 17 (sub7) was present at week 39. Subpopulations with more than one resistance mutation were never seen. Throughout the entire treatment history the dominant subpopulation in M04008 was a wild type virus variant (Figure 5, sub4), although there was an apparent but not statistically significant increase in the overall K103N mutant frequency during the course of treatment.

As in macaque M03250, the V75L and L214F mutations were observed in M04008. V75L first appeared at week 13 (Additional file 2) and was found in 79% of subpopulations present at this time point. At week 17, 81% of subpopulations had the V75L mutation and by week 39, all the subpopulations contained V75L (Additional file 2). In this animal 214L was somewhat more stable than in M03250, and we only observed 5% 214F at week 17 and 34% 214F at week 39.

### Subpopulation dynamics in macaque M04007

**Figure 6:**
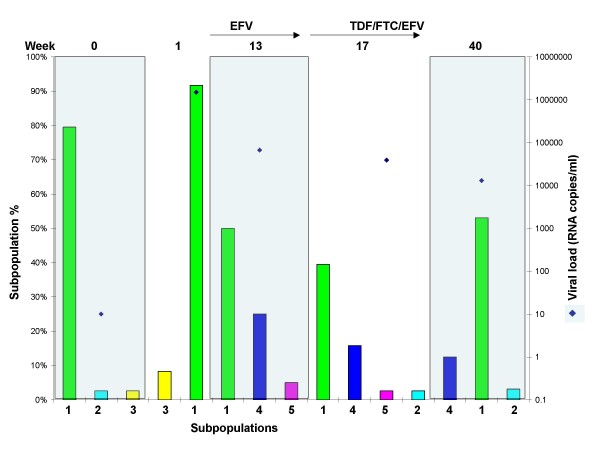
**Dynamics of major subpopulations in macaque M04007**. The major subpopulations present at the weeks shown on the x-axis are represented by colored bars. Subpopulations with identical sequences have the same number and color and drug resistant mutations are labeled. The left y-axis shows the percentage of a subpopulation in the total population. The right y-axis shows the viral load (diamond symbols) for the whole population of each week. Treatments are shown with arrows at the top of the figure along with the week post-infection (week 0 denotes the RT-SHIV challenge stock). Horizontal red bars show the percentage of all subpopulations with a single K103N mutation in the population of each week. Identical subpopulation were given the same subpopulation designation and color code.

Although M04007 received the same ART treatment as M03250 and M04008, no drug resistance mutations were detected in this animal following the short-course EFV monotherapy or during ART. The original wild type dominant subpopulation (sub1, Figure 6) instead persisted as the dominant subpopulation throughout the observation period. This dominant subpopulation was present at a frequency of 90% at week 1 and plateaued at around 50% at subsequent time points. At the same time, another wild type subpopulation emerged in week 13 (sub4, Figure 6) that accounted for about 20% of the total population and persisted at weeks 17 and 40. Several minor subpopulations arose over time (sub3 and sub5, Figure 6), but none had drug resistant mutations and none became dominant. In contrast to macaques M03250 and M04008, only one subpopulation containing V75L was found in M04007 (week 17, 5%) (data not shown), and L214F was not seen at all.

## Discussion

Because of its high mutation rate, large population size, and rapid replication cycle, HIV-1 is able to diversify into a complex genetic population after transmission to a new host. Its pathogenicity in a tractable animal model with a well characterized challenge inoculum, and its sensitivity to widely-used RT inhibitors make the RT-SHIV model a valuable tool for modeling HIV-1 diversity and evolution of resistance to RT inhibitors. Our results showed that RT-SHIV populations in the infected macaques comprised both dominant and minor subpopulations. Similar genetic structures have been revealed by analysis of HIV-1 populations within and between different patient anatomical compartments [22,23]. In most cases, we observed one or two dominant subpopulations and many minor subpopulations in each plasma sample. The dominant subpopulations usually accounted for at least 20% of each virus population.

All three macaques were treated identically, with short course EFV, followed by combination therapy 4 weeks later. Nevertheless, three different patterns of virological response were observed. In M03250, at least 4 subpopulations that encode EFV resistance appeared that contained either of the K103N alleles (AAT or AAC). This occurred following monotherapy. This animal subsequently failed combination therapy, at which time the virus population was characterized by the appearance of viruses with additional mutations, initially K65R (conferring TNF resistance) followed by a clonal subpopulation containing K103N and M184I (conferring FTC resistance), which rapidly became dominant. M04008 had a similar response to the initial monotherapy, with similar proportions of multiple subpopulations containing both AAT and AAC detected by week 17. However, the plasma viremia remained low in this animal, and no new subpopulations containing additional resistance mutations were detected during ART. Macaque M04007, by contrast, contained no subpopulations with drug resistance mutations, despite having been treated identically to the other two animals. M02350 had a much higher viral load than the other two macaques prior to therapy. Although the other two animals had similar viral loads at the time of monotherapy, M04008 had much higher viremia (more than 1 log) at weeks 2 to 10 post-infection. We hypothesize that the patterns observed reflect the relative population sizes of productively infected cells in these animals, with higher viremia in M03250 correlating with the presence and selection of multiple subpopulations of K103N variants by EFV monotherapy and the appearance of additional drug resistance mutations (K65R and M184I/V) within the population containing one of the K103N alleles. The amount of virus replication prior to week 13 in M04007 may have been too small for any K103N mutants to be present in the replicating population at that time or the frequency of K103N in week 13 was too low to be detected with our sampling size. However, this mutation was also not detected by allele specific real-time PCR (ASP) assay [17].

Subpopulations with both K103N alleles were present as early as one week after treatment with EFV in M03250 and M04008. These subpopulations comprised about 20% of the total virus population, as detected by allele specific PCR [17]. However, none of these subpopulations increased in frequency and persisted as a stable subpopulation like the dominant wild-type subpopulation (sub3 in Figure 3) during combination therapy. This persistent stability indicated that a single drug resistance mutation either does not confer a significant selective advantage under this condition or a potential reduced replicative capacity caused by the drug resistance mutation [1,24] allowed additional compensatory mutations to accumulate. For example, in M03250 at weeks 23 and 25, 6-8 weeks after initiation of combination therapy, 11 of 23 minor subpopulations contained K103N mutations and totaled 25-30% of the entire population. A similar phenomenon was observed in monkey M04008, which did not fail therapy. Therefore, even during drug treatment, when virus replication was suppressed, the dominant subpopulations were still wild type. For the most part, there was little change in the composition of the subpopulations during ART in the three animals. Prior to therapy failure, the dominant subpopulations were wild type even during ART. This analysis is supported by other reports, indicating that wild type virus may be preserved during therapy and reemerges after selective pressure is stopped (4)

The presence of a variety of RT-SHIV subpopulations containing K103N in M02350 and M04008 following EFV monotherapy (Additional files 1 and 2), which never became dominant, indicates that K103N alone did not confer a growth advantage to the virus in either the presence or absence of therapy. The existence of multiple minor subpopulations carrying either K103N AAC or AAT suggests that different subpopulations acquired them independently, rather than from a common ancestor, implying that a large effective population size must have been present pre-therapy. By contrast, the outgrowth of a single clonal subpopulation resistant to both EFV and FTC that resulted in therapeutic failure implies that the K103N population may have been so small that the M184I variant was present at a low frequency at the time of initiation of combination therapy. Similarly, a singe clonal population containing both K103N and K65R was present only briefly during combination therapy.

Remarkably, before the doubly resistant population became dominant in macaque M02350, a wild type subpopulation (sub4, Figure 3) present at low frequency before week 22 became the dominant species. Indeed its growth in the population between weeks 23 and 24 was both in relative terms (about 18 to 50% of all populations) and in absolute terms (1620 to 33810 c/ml, Figure 4), indicative of replication and not simply due to population shift. This subpopulation then declined rapidly (at least relatively) to 5% at week 25 and 2% at week 26. It could be that there were beneficial features not directly involved in drug resistance in this variant. Understanding the reason for this phenomenon will await further experimentation.

All monkeys in this study were inoculated with a cell culture supernatant containing RT-SHIV, which was a mixture containing a dominant subpopulation that accounted for 80% of the virus in the challenges. In the two monkeys, M03250 and M04008, this dominant cell supernatant subpopulation was rapidly replaced by new dominant subpopulations (Figures 3 and 5) characterized by the V75L mutation not detected in the inoculum. In M04007, the dominant cell supernatant subpopulation persisted throughout the study (Figure 6). The different fates of the challenge virus within the different animals are perhaps due to differences in host genetics or immunity. V75 is polymorphic in untreated HIV-1 infected patients and it has been suggested that its side chain stabilizes the fingers domain of RT and that its peptide backbone interacts with single-stranded DNA templates [25]. It was also reported in other macaque RT-SHIV studies [26,27], without quantitative analysis. While V75T causes resistance to dideoxyribonucleoside RT inhibitors [28], V75L has not been reported to be a drug resistance mutation. It has, however, been implicated as a secondary mutation for quinoxaline (an NNRTI inhibitor) in vitro [29]. V75L appeared in that study after the introduction of the quinoxaline resistant mutation G190Q. In our study, V75L appeared before the emergence of any drug resistant mutations, and it spread to almost all subpopulations in later time points. This pattern suggests that V75L probably conferred a selective advantage to the virus on its own, rather than being secondary to known drug resistant mutations. In M04007 V75L was not detected at week 13 and the only V75L subpopulation found in week 17 did not persist or spread to other subpopulations at later time points, suggesting that the selective advantage it confers may be host specific. Since ultradeep sequencing showed that this mutation was present at less than 0.01% of the genomes in the inoculum, it must have arisen de novo and been selected in all three macaques. V75 has been shown to be within a human A3 supertype CTL epitope (Los Alamos HIV Immunology Database). Further studies are needed to investigate if this mutation is also within a macaque CTL epitope.

We observed a rapid increase in the frequency of another common polymorphism, L214F, in M03250 from 0% at week 0 to 41% at week 10, and 100% at week 25. The frequency of 214F increased much more slowly in M04008, and 214F was not observed at all in M04007. The 214F mutation is associated with nucleoside analogue mutation cluster 2 (D67N+K70R+K219Q+T215F) and negatively associated with nucleotide analogue mutation cluster 1 (M41L+L210W+T215Y) [30,31]. Our data indicate that 214F might be associated with a negative virological response to NNRTI treatment because of its low frequency in M04008 and M04007, which responded well to the NNTRI treatment, and its rapidly increasing frequency in M03250, which failed the treatment. L214F was reported in previous RT-SHIV studies [26,27], although no quantitative analysis was reported.

## Conclusion

This study quantitatively describes virus evolution and population dynamics patterns in an animal model. Our quantitative approach of viral population dynamics allows us to observe the relative competitiveness of different viral variants prior to and during antiretroviral treatment. Our results imply that RT-SHIV in infected macaques provides a valuable model for understanding the shifting patterns of HIV subpopulations in infected humans and the roles played by factors including population size, selection and drift, and antiviral therapy. Further studies will be needed to determine how well this model recapitulates the behavior of HIV-1 in patients treated with ART.

## Methods

Three pigtail macaques that were housed at the Washington National Primate Research Center according to American Association for Accreditation of Laboratory Animal Care standards were infected intravenously with 10^5 ^infectious units of RT-SHIV_mne _[16,17]. The macaques were treated orally with 200 mg EFV (Sustiva; Bristol Myers-Squibb, Princeton, NJ) on days 1, 2, and 4 at 13 weeks post-infection. The animals subsequently received daily ART consisting of TNF (20 mg/kg, subcutaneous; Gilead Sciences, Foster City, CA), FTC (50 mg/kg, subcutaneous; Gilead), and EFV (200 mg, oral) for 20 weeks beginning at week 17. Plasma samples were collected weekly throughout the study.

SGS was used to sequence the viral RNA. Briefly, viral RNA was extracted for cDNA synthesis as described previously [32,33]. To obtain PCR products for SGS, the cDNA was diluted until approximately 30% of the PCR reactions yielded DNA product. cDNA was added to the PCR mix containing primers 2195F (5' AAA CAA TGG CCA TTG ACA GAA GA 3') and 2818R (5' CCA AAG GAA TGG AGG TTC TTT CTG 3'), and then nested PCR primers B2203F (5' ATG GCC ATT GAC AGA AGA AAA AAT 3') and B2814R (5' AGG AAT GGA GGT TCT TTC TGA TGT TT 3') to amplify a 620 nucleotide fragment of HIV-1 RT. In most cases, more than 30 sequences were obtained from each sample. Sequence subpopulation analyses were performed using an in-house computer program written in Perl scripting language (available upon request) and using MEGA 4 [34]. Sequences obtained from each plasma sample were compared to identify unique subpopulations. A unique subpopulation was defined as one or more virus genome fragments of identical sequence. The dominant virus subpopulation was defined as the subpopulation containing the largest number of sequences at each time point. Subpopulations from each sample were compared to analyze the population dynamics of HIV-1 RT prior to and during ART. Drug resistant mutations were identified based on Stanford HIV Drug Resistance Database definitions http://hivdb.stanford.edu.

## Competing interests

The authors declare that they have no competing interests.

## Authors' contributions

WS conceived the concept, performed data analyses, and wrote the manuscript. MK conducted SGS, contributed to the discussion of the study and to the writing and review of the manuscript. FM, JWM, RMS, contributed to the discussion, writing and review of the manuscript. JDL, VNK, ZA developed the RT-SHIV macaque model, shared plasma samples for the study, and contributed to the reviewing and discussion of the manuscript. JMC contributed to the refinement of the concept, the discussion, the writing and the review of the manuscript. SEP contributed to the refinement of the concept, the discussion, the writing and the review of the manuscript, and coordinated the project.

## Supplementary Material

Additional file 1 **Supplemental Table S1**. Selected subpopulations shown in Figures 3 and 4 and subpopulations containing drug resistance mutations from animal M03250. Click here for file

Additional file 2 **Supplemental Table S2**. Selected subpopulations shown in Figure 5 and subpopulations containing drug resistance mutations from animal M 04008. Click here for file
