# Supplementary material for: RT-SHIV subpopulation dynamics in infected macaques during anti-HIV therapy
Source: Retrovirology. 2009 Nov 4;6:101. doi: 10.1186/1742-4690-6-101 (PMC2776578; doi:10.1186/1742-4690-6-101)
Supplement: Additional file 1 — Supplemental Table S1. Selected subpopulations shown in Figures 3 and 4 and subpopulations containing drug resistance mutations from animal M03250. [file 1742-4690-6-101-S1.doc]

Supplemental Table 1. Selected subpopulations shown in Figures 3 and 4 and subpopulations containing drug resistance mutations from animal M03250. This table contains only the subpopulations shown in Figure 3 and additional subpopulations containing the K103N mutation.

^1^ Only non-synonymous mutations are listed for each subpopulation.

^2^ The column labeled “% K103N” shows the percentage of subpopulations with a single K103N mutation (without linkage to another drug resistance mutation) in the population of each week.

^3^The column labeled “% L214F” shows the percentage of subpopulations with 214F mutation in the population of each week. Only selected subpopulations listed in this table.

^4^The column labeled “% DRM” shows the percentage of subpopulations with any drug resistant mutations including K65R, K103N, M184I, and M184V linked or not linked.
